# Adherence to adjuvant endocrine therapy in breast cancer: a single regional Australian centre experience

**DOI:** 10.3389/fonc.2026.1704927

**Published:** 2026-02-23

**Authors:** Dianheng Bu, Arvind Sahu, Javier Torres

**Affiliations:** 1 Department of Medical Oncology, Goulburn Valley Health, Shepparton, VIC, Australia; 2 Department of Rural Health, Shepparton Clinical School, The University of Melbourne Melbourne Medical School, Melbourne, VIC, Australia

**Keywords:** breast cancer, endocrine therapy, medication adherence, rural oncology, side effect

## Abstract

**Introduction:**

Adjuvant endocrine therapy reduces cancer recurrence and improves overall survival in hormone receptor–positive breast cancer. Maintaining long-term adherence to endocrine therapy can be challenging, and non-adherence has been associated with higher recurrence rates and reduced survival. Despite its importance, data describing adherence patterns in regional Australian populations are limited. This study aimed to assess adherence to adjuvant endocrine therapy in a regional Australian cancer center and to examine factors associated with non-adherence.

**Methods:**

A cross-sectional survey was conducted at a regional Australian cancer center between March 2023 and July 2024. Eligible participants were women receiving adjuvant oral endocrine therapy for breast cancer. Adherence was assessed using the six-item Simplified Medication Adherence Questionnaire (SMAQ). Participants also reported treatment-related side effects.

**Results:**

One hundred women were included, with a median age of 62.5 years. The adherence rate as determined by the SMAQ was 65%. Participants reported an average of 2.5 treatment-related side effects, with hot flashes being the most commonly reported (59%). Both the presence and number of reported side effects were associated with lower adherence. In multivariable analyses, a higher side-effect burden remained associated with adherence after adjustment for other covariates.

**Discussion:**

This study demonstrates suboptimal adherence to adjuvant endocrine therapy in a regional Australian cohort and highlights treatment-related side effects and forgetfulness as key barriers to adherence. These findings underscore the importance of proactive side-effect management and practical adherence support strategies within regional cancer care settings.

## 1 Introduction

Breast cancer is the most prevalent cancer among females worldwide, accounting for 11.6% of all cancer diagnoses (1). Credited to the improvement in breast cancer screening, the majority of breast cancer in Australia is non-metastatic (stage I-III) at diagnosis (2). Breast cancers are classified based on their receptor status, with hormone receptor-positive disease accounting for approximately 70-75% of all breast cancers (3). The management of early and locally advanced hormone receptor-positive breast cancer involves adjuvant endocrine therapy with either a selective estrogen receptor modulator (SERM) such as tamoxifen or an aromatase inhibitor like letrozole or exemestane. Completion of adjuvant endocrine therapy is associated with reduced cancer recurrence and improved overall survival (4–7).

Medication adherence is defined by the WHO as the extent to which a patient follows the medication regimen agreed with their healthcare provider (8–10). Medication adherence can be assessed objectively by measuring drug or metabolite concentration in body fluids (9, 11). It can also be evaluated through prescription data by calculating the medication possession ratio (MPR), which is the proportion of days a patient has medication available during a particular period (9, 11). Alternatively, medication adherence can be assessed subjectively using patient interviews or self-report questionnaires (9).

In the setting of adjuvant endocrine therapy for breast cancer, a systematic review demonstrated that non-adherence to endocrine therapy negatively affects event-free and overall survival (12). Long-term adherence to adjuvant endocrine therapy is challenging, as it is a medication that requires daily dosing for as long as 10 years with extended therapy in high-risk patients (13).

Furthermore, while endocrine therapies are crucial in preventing cancer recurrence, non-adherence to treatment does not result in any short-term adverse symptoms. Hence, due to the less immediate perceived benefits, adherence to preventive medications is lower (14). Although generally well tolerated, many patients experience side effects, including hot flashes, fatigue, and arthralgia (15, 16). Adherence rates are suboptimal, ranging from 41–72% in a systematic review (17).

Medication adherence in regional and rural settings may be influenced by several factors compared with metropolitan populations, including higher proportions of patients with lower socioeconomic status, reduced access to oncology and allied health services, increased travel time to appointments and pharmacies, greater financial burden, and unmet psychological support needs (18–20). Together, these patient and service-related constraints may adversely affect adherence to long-term therapies such as adjuvant endocrine treatment, which require sustained engagement over many years. Consistent with this, two studies comparing adherence to adjuvant endocrine therapy in rural and metropolitan areas demonstrated lower adherence rates in rural centers (21, 22). Despite well-established real-world evidence describing factors associated with adherence to adjuvant endocrine therapy, there are limited real-world data on patterns of adherence among patients with early breast cancer treated in regional Australian oncology services (17).

The primary aim of this study was to assess adherence to adjuvant endocrine therapy among women with hormone receptor-positive breast cancer treated at a regional Australian cancer centre. Secondary aims were to explore associations between patient, treatment, and side-effect-related factors and adherence.

## 2 Materials and methods

### 2.1 Participants and recruitment

This cross-sectional survey included women with hormone receptor-positive breast cancer who underwent adjuvant endocrine therapy for breast cancer at a regional Victorian hospital. Eligible participants were recruited by the study team during their oncology clinic appointments to complete a survey regarding adherence to their endocrine therapy. Each participant was allowed to complete the survey once during the entire recruitment period. A formal sample size calculation was not undertaken. The target sample size of 100 was chosen pragmatically, based on anticipated recruitment feasibility within the study period. Consecutive consenting participants were enrolled until this target was reached.

### 2.2 Inclusion and exclusion criteria

Inclusion criteria were (1): female patients aged ≥18 years (2); histologically confirmed hormone receptor–positive breast cancer; and (3) receipt of adjuvant endocrine therapy for a minimum duration of 3 months prior to survey administration.

Patients were excluded if they had metastatic disease at diagnosis, were receiving endocrine therapy for non-adjuvant indications (including the neoadjuvant setting), declined participation, or had previously participated in the study, as only one survey response per individual was permitted. Patients who had discontinued endocrine therapy prior to survey administration were also excluded.

### 2.3 Survey tool

The paper survey required participants to fill out basic demographic information, details regarding their cancer management, and the duration and side effects experienced regarding their endocrine therapy.

Adherence to endocrine therapy was examined using the Simplified Medication Adherence Questionnaire (SMAQ), which is a self-report scale containing six questions. It was originally developed to assess adherence to antiviral therapy in HIV patients and has since been demonstrated to have adequate reliability and validity for assessing adherence in other medications such as tacrolimus for renal transplant patients and antihypertensives (23–25). Furthermore, it has been used in several previous studies to evaluate adherence to oral endocrine therapy in breast cancer (26–29). One study found moderate concordance between adherence assessed by SMAQ to plasma drug concentration, prescription refill and physician-rated adherence (28).

The following six questions were asked in the SMAQ:

1. Do you ever forget to take your medicine? (Response: yes-no),
2. Are you careless at times about taking your medicine? (Response: yes-no),
3. Sometimes if you feel worse, do you stop taking your medicines? (Response: yes-no),
4. Think back to last week. How often did you not take your medicine? (4-point Likert scale: from “Never” to “5-7 times”).
5. Did you fail to take any of your medicine over the past weekend? (Response: yes-no).
6. Over the past three months, how many days did you not take any medicine at all?

The SMAQ classifies a patient as non-adherent if they respond “yes” to any of questions 1–3 or 5, miss more than two doses in the past week, or have more than two days of complete non-medication intake over the past three months (23).

### 2.4 Data management and analysis

The results from the completed paper surveys were recorded on Microsoft Excel and later transferred and analyzed on IBM SPSS Statistics for Windows Version 29.0 (Armonk, NY: IBM Corp.). The adherence rate is reported as a percentage of all participants. Univariate analyse using t-test and chi-square were conducted to identify factors associated with non-adherence with a significant alpha level set at p < 0.05. Given the relatively small sample size of the study, a parsimonious multivariable logistic regression model was constructed to examine factors associated with adherence while balancing the risk of overfitting. Variables demonstrating statistically significant associations in univariable analyses were included in the multivariable model. Collinearity between covariates included in the final model was assessed using variance inflation factors (VIFs).

### 2.5 Ethics

Ethical approval was received from the Goulburn Valley Health Human Research Ethics Committee in 2023. Participants read through an explanatory page and were given the opportunity to ask the research team questions prior to signing the informed consent form and completing the survey.

## 3 Results

### 3.1 Patient demographics

Between March 2023 and July 2024, 100 female patients undergoing adjuvant oral endocrine therapy at our institution participated in the survey. The median age was 62.5 years (IQR: 51.8–62.9), and the median duration of therapy was 2 years. The majority of women had an Eastern Cooperative Oncology Group (ECOG) performance status score of 1. Regarding disease stage, 42 patients had stage I, 42 had stage II, and 16 had stage III breast cancer. Most patients received letrozole (36), followed by anastrozole (20), exemestane (22), and tamoxifen (22) (**Table 1**).

**Table 1:** Demographics (N = 100).

Variables	Category	n (%)
Age, years		
	Median (Range)	62.5 (31–89)
ECOG performance status score-no.		
	0	36 (36.0)
	1	52 (52.0)
	2	9 (9.0)
	3	3 (3.0)
Overall disease stage-no.		
	Stage I	42 (42.0)
	Stage II	42 (42.0)
	Stage III	16 (16.0)
Nodal involvement – no.		
	Negative	61 (61.0)
	Positive	39 (39.0)
Unilateral vs Bilateral breast cancer -no.		
	Unilateral	97 (97.0)
	Bilateral	3 (3.0)
Previous chemotherapy-no.		
	Yes	46 (46.0)
	No	54 (54.0)
Previous radiation therapy-no.		
	Yes	80 (80.0)
	No	20 (20.0)
Type of breast surgery -no.		
	Lumpectomy	60 (60.0)
	Mastectomy	40 (40.0)
	

### 3.2 Side effect profile

**Figure 1:**
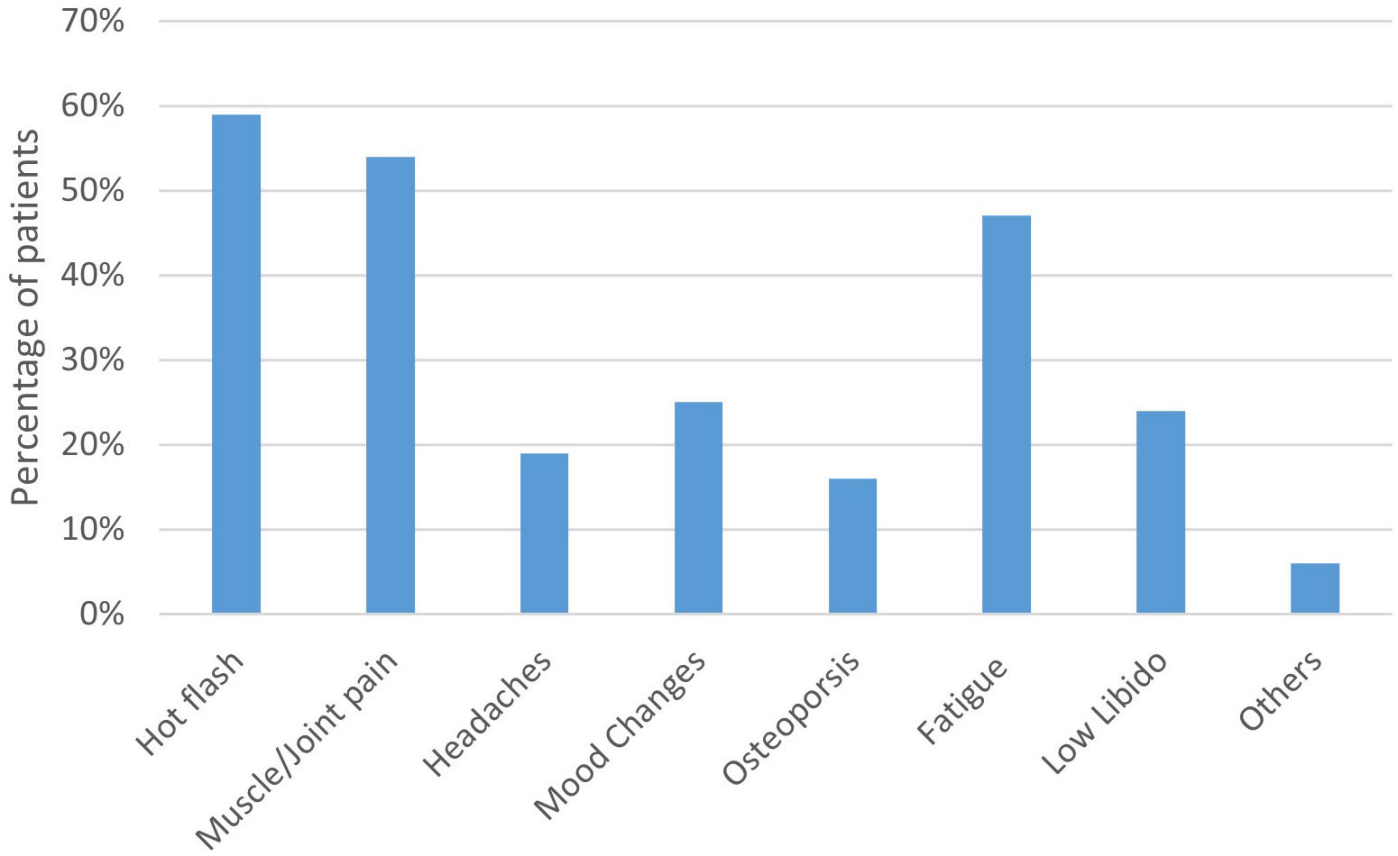
Frequency of self-reported endocrine therapy–related side effects among patients.

Patients experienced an average number of 2.5 side effects while on treatment. The most commonly reported side effect were hot flashes (59%), followed by muscle/joint pain (54%) and fatigue (47%). In terms of side effects experienced on different medications, patients receiving exemestane reported a higher mean number of side effects (4.1) compared with the other three medications (P<0.001) (**Figure 1**).

### 3.3 Adherence rate and associated factors

The adherence rate for oral endocrine therapy in our cohort as determined by the SMAQ, was 65%. ECOG performance status, disease stage, and duration of endocrine therapy were not significantly associated with adherence. Patients who had cessation of previous endocrine therapy due to intolerance of side effects, their adherence rate of 60.0% on the current therapy demonstrated no statistically significant difference to the rest of the participants at 65.8% (**Table 2**).

**Table 2:** Responses to simplified medication adherence questionnaire (SMAQ) (N = 100).

Questions	Response	n (%)
Do you ever forget to take you medicine?		
	Yes	31 (31.0)
	No	69 (69.0)
Are you careless at times about taking your medicine?		
	Yes	9 (9.0)
	No	91 (91.0)
Sometimes if you feel worse, do you stop taking your medicines?		
	Yes	18 (18.0)
	No	82 (82.0)
Think back to last week. How often did you not take your medicine?		
	Never	88 (88.0)
	1-2 Times	9 (9.0)
	3-5 times	1 (1.0)
	5-7 times	2 (2.0)
Did you fail to take any of your medicine over the past weekend?		
	Yes	5 (5.0)
	No	95 (95.0)
Over the past three months, on how many days did you not take any medicine at all?		
	0	66 (66.0)
	1-4 days	20 (20.0)
	5-10 days	11 (11.0)
	11-20 days	2 (2.0)
	20+ days	1 (1.0)
Adherence to medication as per SMAQ criteria		
	Yes	65 (65.0)
	No	35 (35.0)

The age of patients, the number of side effects and types of medication were significantly associated with adherence. In our cohort, exemestane was associated with the lowest adherence rate at 40.9% (p=0.042), compared to anastrozole and letrozole, both at 75%, and tamoxifen at 63.6%. The non-adherent group was younger with a mean age of 55.2 compared to 65.0 in the adherent group (p<0.001). They experienced an average of 4.2 side effects which was 2.6 more than the adherent group (p<0.001). Regarding specific side effects, the presence of each side effect was associated with higher rates of non-adherence (**Table 3**).

**Table 3:** Percentage of patients experiencing side effects in the adherent and non-adherent group.

Side effects	Adherent (%)	Non-Adherent (%)	P-Value
Hot Flash	49.2	77.1	0.007
Muscle/ Joint Pain	38.5	82.9	<0.001
Headache	7.7	40	<0.001
Mood changes	15.4	42.9	0.002
Osteoporosis	9.2	28.6	0.012
Fatigue (%)	47	85.7	<0.001
Low libido (%)	13.8	42.9	0.001
Others (%)	0	17.1	<0.001

The SMAQ includes the question, ‘Sometimes if you feel worse, do you stop taking your medicines?’ which explores intentional non-adherence in the context of side effects. Among the 18 patients who answered “yes” to this question, they experienced on average 4.9 side effects, with 17 (94.4%) patients reported hot flashes or fatigue, 14 (77.9%) muscle/joint pain, 10 (55.6%) low libido, 9 (50%) headaches and mood changes, all of which were significantly higher than those reported by patients who answered “no”.

### 3.4 Multivariate analysis of factors associated with adherence

In the univariate analysis, three variables including the age of patients, type of medication and number of side effects were significantly associated with adherence. However, in multivariate analysis of the three variables with logistic regression, after adjusting for other variables, only the number of side effects remains independently associated with adherence (B = -0.868, p < 0.001) with an odds ratio of 0.420 (95% CI: 0.282 - 0.624). Collinearity diagnostics did not indicate problematic multicollinearity, with all variance inflation factors below 2 (**Supplementary Table S1**).

## 4 Discussion

### 4.1 Adherence in the context of existing literature

Our study cohort reported an adherence rate of 65% to oral endocrine therapy in the adjuvant breast cancer setting, as assessed by the SMAQ. This adherence rate is similar to or lower than other studies that assessed adherence to adjuvant endocrine therapy with SMAQ, with a reported adherence rate ranging from 53.3% to 86.2% (26, 27, 29). More broadly, a systematic review of 30 studies reported adherence rates between 41-72% across studies using a variety of assessment methods (17). Non-adherence to treatment has been associated with poorer disease-free survival and overall survival (12). It is imperative to improve adherence to therapy to maximize the benefits of oral endocrine medication.

### 4.2 Forgetfulness and reminder-based interventions

Across all studies assessing adherence with SMAQ, forgetfulness emerges as the most prevalent barrier to adherence (26, 27, 29). In our study, 31% of all participants reported forgetting to take their endocrine therapy. One randomized control trial explored whether text reminders to take oral endocrine therapy can improve adherence (29). Although text was perceived as acceptable, easy to understand and useful for patients, it did not improve adherence assessed by SMAQ, nor did it result in higher androstenedione levels at 1 year (29). However, a meta-analysis of 16 RCTs on text message reminders to improve chronic disease medication adherence found an absolute increase in adherence of 17.8% (30). Hence, further evidence is required to determine if text messages could be effective in improving adherence in the context of oral endocrine therapy. Another commonly used reminder system to improve adherence is packaging interventions, such as pill boxes and Webster packs, which have been shown to enhance adherence to long-term oral medication (31, 32). A cohort study where all patients received prescriptions through blister packs led to a high adherence rate of 97% defined as an 80% or higher medication possession ratio (33).

Furthermore, the involvement of health care professionals such as breast care nurses can serve as an effective reminder system. A randomized study based in rural Ethiopia demonstrated that involvement of breast care nurses who deliver support, patient education and medicine-reminder phone calls has led to an adherence rate of 70% measured with SMAQ compared to 44.8% in the control group (p=0.036) (26). This study emphasizes the importance of breast care nurses in improving adherence, especially in regional areas where there is a shortage of oncologists.

Taken together, addressing forgetfulness as a barrier to medication adherence is likely to require a multimodal approach incorporating medication packaging services, text message reminders, nurse-led follow-up, and reminder phone calls. Given the resource limitations frequently encountered in regional settings, breast cancer treatment teams should aim to direct patients toward accessible and acceptable supportive services to maximise adherence to adjuvant endocrine therapy.

### 4.3 Associations between treatment-related side effects and adherence

Our study found that participants who reported more side effects had lower adherence rates. In our study cohort, the non-adherent group reported an average number of 4.2 side effects compared to 1.6 side effects from participants who adhered to therapy. Furthermore, 18% of participants demonstrated intentional non-adherence and they responded that they stopped taking medication when they felt worse from side effects. This association is further reinforced by the result that the presence of each side effect is associated with a lower percentage of patient adherence to therapy. This suggests the potential importance of promptly addressing side effects as part of adherence support. A systematic review of eleven studies focusing on side-effect management through consultations with an oncology care team revealed mixed results, with six studies demonstrating a significant improvement in adherence after addressing treatment-related side effects (34). Our study found that for patients who switched endocrine therapy due to intolerance, adherence to the new medication was not significantly different from that of patients who did not require a medication change. These findings highlight the importance of addressing treatment-related side effects to support adherence. In clinical practice, proactive side-effect assessment at routine follow-up consultations may facilitate early recognition of treatment-related toxicity. Evidence-based pharmacologic and non-pharmacologic strategies, together with patient education regarding expected side effects, have been shown to alleviate common endocrine therapy–related symptoms such as hot flashes, sexual dysfunction, and musculoskeletal symptoms (35). Furthermore, consideration of switching endocrine therapy for patients with persistent intolerance may represent a practical strategy to reduce intentional non-adherence and support sustained treatment continuation. In this context, our study observed an association between exemestane use, higher reported side-effect burden, and lower adherence rates. These findings should be interpreted cautiously. In multivariable analysis adjusting for age and number of reported side effects, exemestane was no longer independently associated with adherence. This analysis did not adjust for line of therapy, and no stratification was performed according to prior tamoxifen exposure. Accordingly, these results should be considered hypothesis-generating and should not be interpreted as evidence that exemestane is intrinsically less tolerable or associated with poorer adherence.

### 4.4 Study limitations

Several limitations were identified in this study. The study included a relatively small, single-center cohort of 100 participants, with the sample size determined pragmatically based on recruitment feasibility within the study period and without a formal *a priori* sample size calculation. This may limit the ability to detect smaller effect sizes and reduce the generalizability of the findings. Accordingly, this study should be considered exploratory in nature. Due to the small sample size, a parsimonious multivariable approach was used; although collinearity diagnostics did not suggest problematic multicollinearity, residual confounding and omitted-variable bias remain possible, and adjusted estimates should be interpreted cautiously. In addition, this was a single-center study conducted in a regional setting. Regional cancer centers in Australia may differ substantially in terms of healthcare accessibility, degree of rurality, population characteristics, and socioeconomic status, all of which can influence access to medications and cancer care, and in turn medication adherence. As a result, the findings from this single cohort may not be generalizable to all patients receiving adjuvant endocrine therapy across regional Australia. Furthermore, as this study was cross-sectional in design, it was not possible to assess changes in adherence over time, and the observed findings should be interpreted as associations rather than causal relationships. Additionally, only the presence of certain side effects was measured, without considering their severity or impact on daily functioning, which may influence adherence to varying magnitudes. Comparisons between endocrine agents were not adjusted for line of therapy or prior treatment exposure and are therefore subject to treatment sequence bias. The adherence assessment tool (SMAQ) was originally developed for antiviral therapy in HIV patients; although it has been used to assess adherence to adjuvant therapy in breast cancer, its validity and reliability have not been formally evaluated in this setting. Adherence and side effects were self-reported using SMAQ and are therefore subject to recall and social desirability bias. Consequently, the observed adherence rate of 65% is likely an overestimation of true adherence. Misclassification of non-adherent individuals as adherent due to self-report may attenuate observed associations. In addition, SMAQ assesses self-reported medication-taking behaviour rather than sustained treatment persistence, which should be considered when interpreting adherence estimates.Moreover, the survey was unable to capture several potential confounders that may influence adherence beyond the collected demographic variables, including socioeconomic status, comorbid medical conditions, distance to oncology services or pharmacies, and access to supportive care services.

## 5 Conclusion

Our cross-sectional study found suboptimal adherence to adjuvant oral endocrine therapy for hormone receptor-positive breast cancer, which is consistent with existing literature. Improving adherence remains an ongoing challenge for breast cancer clinicians aiming to maximize the therapeutic benefit of adjuvant endocrine therapy. Forgetfulness and avoidance of side effects were the primary barriers to adherence identified in our study cohort. These findings highlight the potential value of multifaceted, practical approaches delivered by the multidisciplinary care team to address barriers to medication adherence in regional cancer care settings, including the use of acceptable reminder systems, patient education, and proactive management of treatment-related side effects.

## Data Availability

The original contributions presented in the study are included in the article/**Supplementary Material**. Further inquiries can be directed to the corresponding author.
