# Supplementary material for: Adherence to adjuvant endocrine therapy in breast cancer: a single regional Australian centre experience
Source: Front Oncol. 2026 Feb 23;16:1704927. doi: 10.3389/fonc.2026.1704927 (PMC12967936; doi:10.3389/fonc.2026.1704927)
Supplement: Supplementary file 1 [file Table1.docx]

Supplementary Table S1. Variance inflation factors for variables included in the multivariable model

| **Variable** | **VIF** |
| --- | --- |
| Age | 1.52 |
| Endocrine agent | 1.06 |
| Number of side effects | 1.49 |
